# Getting the Show on the Road! States Amp Up a Life Course Approach to Alzheimer's

**DOI:** 10.1093/geroni/igaa057.2543

**Published:** 2020-12-16

**Authors:** John Shean, Molly French, Carissa Beatty

**Affiliations:** 1 Alzheimer's Association, Washington, District of Columbia, United States; 2 Emory Centers For Training And Technical Assistance, atlanta, Georgia, United States

## Abstract

Alzheimer’s and other dementias are chronic conditions that disproportionately impact some populations. The dementia continuum spans decades, so life-course approaches by state health departments (SHDs) are crucial. SHDs’ implementation of the Healthy Brain Initiative Road Map (RM) represents policy/system/environmental actions to improve population-level dementia outcomes. METHODS. Alzheimer’s Association uses online reporting by affiliate chapters to monitor SHDs’ RM implementation. Association staff reviewed data and calculated frequencies by RM action and issue areas. Documentation from SHDs and national partners are supplementary data. RESULTS. Preliminary results from July-December 2019 indicate 34 SHDs were implementing 60 RM actions. Interventions clustered in risk reduction, early detection, workforce competencies, surveillance, and emergency preparedness. RM implementation lagged on access to community supports, attention to caregiving, and workforce supply analyses. A majority of states had multi-faceted initiatives. IMPLICATIONS. SHDs are deploying life-course approaches for dementia. SHD leaders must build towards a full response by expanding RM implementation.

